# Commentary: Montelukast Prevents Mice Against Acetaminophen-Induced Liver Injury

**DOI:** 10.3389/fphar.2019.01289

**Published:** 2019-10-25

**Authors:** Ralf Weiskirchen

**Affiliations:** Institute of Molecular Pathobiochemistry, Experimental Gene Therapy and Clinical Chemistry (IFMPEGKC), RWTH University Hospital Aachen, Aachen, Germany

**Keywords:** liver, animal model, Montelukast, acetaminophen, APAP, Singulair, GSH/GSSH, therapy

Montelukast sodium (CAS-No.: 151767-02-1), marketed as Singulair, acts as a selective orally active leukotriene receptor antagonist inhibiting the cysteinyl leukotriene receptor 1 (CysLTR1). It is a medication for treatment of exercise-induced asthma, bronchospasm, allergic rhinitis, and was applied in some patients as a beneficial add-on remedy in the therapy of chronic idiopathic urticaria. Molecularly, this drug selectively antagonizes the activities of the cysteinyl leukotrienes LTC_4_, LTD_4_, and LTE_4_, thereby displaying important anti-oxidative and anti-inflammatory effects in various tissues and organs (Singh et al., 2013). Recent work conducted in fibroblast-like synoviocytes, which are the main contributors in rheumatoid arthritis, has shown that Montelukast attenuated IL-1β-induced phosphorylation and degradation of IκBα, nuclear translocation of p65 and NF-κB activity (Dong et al., 2018). These findings supports previous findings showing that Montelukast highly potently prevents the activation of NF-κB signaling, which is a central pathway controlling the inflammatory response (Maeba et al., 2005). In regard to liver, Montelukast treatment improved hepatic fibrosis in rats in a bile duct ligation model by modulating hepatic expression of TGF-β, NF-κB, changing the MMP-9/TIMP-1 ratio, and mediating other hepatoprotective effects (El-Swefy and Hassanen, 2009; Kuru et al., 2015). In humans, the drug is usually well-tolerated and reported adverse drug-reactions are mild and similar in type and frequency to side effects induced by a placebo. However, it is already known for long time that some patients under Montelukast therapy occasionally developed bleeding tendency, allergic reactions, hepatitis, and joint pain, muscle aches, and muscle cramps (Russmann et al., 2003; Duchemin et al., 2004; Goldstein et al., 2004).

Pu et al. (2019) tested whether the pharmacological inhibition of CysLTR1 by Montelukast impacts acetaminophen (APAP)-induced acute liver failure in C57BL/6J mice. APAP-induced liver injury is a rather complex process in which intracellular and extracellular events are involved including mitochondrial and endoplasmic reticulum oxidative stress, sterile inflammation, microcirculatory dysfunction and liver regeneration (Yan et al., 2018).

The authors found that Montelukast-induced CysLTR1 expression and significantly prevented drug-induced liver damage when given at 3 mg/kg body weight by gavage 1 h after APAP administration. In addition, the authors demonstrated that Montelukast upregulated hepatic GSH/GSSH level, provoked expression of Glutathione S-transferase A2 (GSTA2), and prevented liver inflammation, oxidative stress, as well as activation of the c-Jun-N-terminal kinase (JNK) (**Figure 1**). Moreover, Montelukast was *in vitro* effective in blocking cell death and inflammation induced by APAP treatment in mouse primary hepatocytes. Based on this data, the authors concluded that the inhibition of CysLTR1 by Montelukast may be a potential treatment strategy in APAP-induced hepatotoxicity.

**Figure 1:**
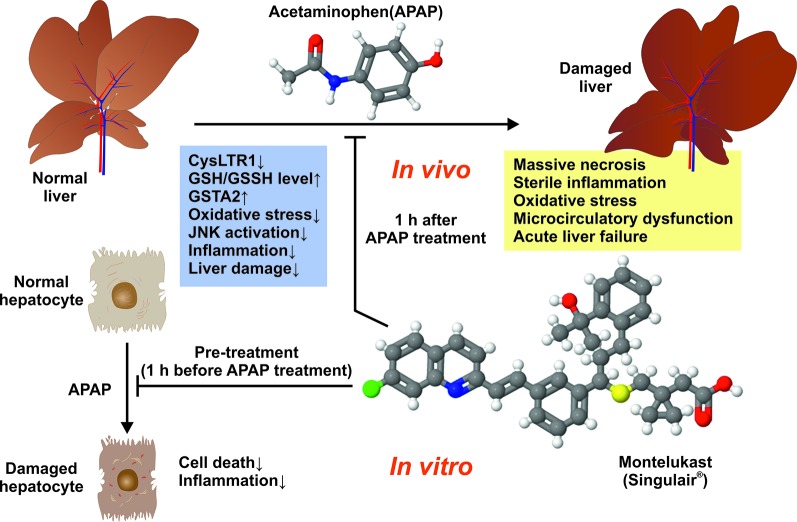
Montelukast prevents mice against APAP-induced liver damage. The observed beneficial effects of Montelukast in APAP-treated mice are depicted.

The fact that targeting the CysLTR1 axis provokes a significant upregulation of hepatic GSH/GSSH level and GSTA2 expression shows that Montelukast increases the potential to scavenge ROS and genes relevant in the detoxification of toxins and products of oxidative stress. In line, the finding that JNK activation is blocked points to the therapeutic potential of Montelukast because the magnitude and duration of JNK activation is known to be a major determinant of acute injury from APAP (Win et al., 2018). The decrease of pro-inflammatory chemokines or cytokines including *Mcp1*, *Tnfα*, *Il*6, and *Il18* by Montelukast in primary cultured hepatocytes treated with APAP, demonstrates that this drug prevents inflammatory signaling.

Of course these findings are highly interesting and add Montelukast to the large list of drugs that evolve beneficial effects on liver health in classical rodent disease models (Weiskirchen, 2016). Commonly, these substances target intracellular reactive oxygen species (ROS) formation, prevent hepatic infiltration with circulating blood cells, or target pro-inflammatory and pro-fibrotic signaling pathways or mediators involved in generation or turnover of extracellular matrix (Weiskirchen, 2016).

However, in most cases, it is highly questionable if the observed therapeutic promise of a drug candidate can be translated to the human situation. Although most of the mechanistic insight in the pathophysiology of APAP hepatoxicity has been gained from experiments with the murine system, the injury process progresses in mice is much faster than in humans (Jaeschke et al., 2014). There are also some limitations in experiments conducted in primary hepatocytes. In culture, these parenchymal cells lack the contact to non-parenchymal cells and are usually exposed to highly artificial media in which numerous alterations in regard to gene expression and oxidative stress will affect the sensitivity to drugs such as APAP (Jaeschke et al., 2014). Moreover, hepatic injury caused by acetaminophen is dependent upon conversion to a toxic and reactive metabolite (*N*-acetyl-*p*-benzoquinone imine, NAPQI) (McGill and Jaeschke, 2013), which depletes hepatic GSH, binds to proteins, and causes severe damage to the liver. Therefore, any intervention that blocks NAPQI formation will also prevent downstream effects, including inflammation. The data presented by the authors shows that Montelukast impacts NAPQI formation. Thus, the major and potentially more important mechanism of protection is inhibition of acetaminophen bioactivation, not inhibition of JNK signaling or inflammation. Therefore, the reduction in inflammation, for example, is likely a secondary effect of the protection.

To sum up, because APAP-induced liver injury is one, if not the most frequently encountered drug hepatotoxicity, it is absolutely necessary to establish solid interventions or identify new therapeutic effective drugs impacting the outcome of APAP-induced liver injury. The study by Pu et al. (2019) adds a new candidate drug showing beneficial effects in ongoing APAP-induced liver injury. It will now be interesting to evaluate the curative effects of Montelukast in the respective rodent model in more detail and to test if the hepatoprotective effects can be reproduced in human volunteers. In addition, it will be essential to understand the cause of hepatotoxicity from Montelukast occurring with rare incidence (Sass et al., 2003; Russmann et al., 2003; Goldstein et al., 2004).

## Author Contributions

RW has written this General Commentary.

## Conflict of Interest

The author declares that the research was conducted in the absence of any commercial or financial relationships that could be construed as a potential conflict of interest.
